# The *DiGEM *trial protocol – a randomised controlled trial to determine the effect on glycaemic control of different strategies of blood glucose self-monitoring in people with type 2 diabetes [ISRCTN47464659]

**DOI:** 10.1186/1471-2296-6-25

**Published:** 2005-06-16

**Authors:** Andrew Farmer, Alisha Wade, David P French, Elizabeth Goyder, Ann Louise Kinmonth, Andrew Neil

**Affiliations:** 1 Department of Primary Health Care, University of Oxford, Old Road Campus, Oxford, OX3 7LF, UK; 2 School of Sport and Exercise Sciences, University of Birmingham, Birmingham, B15 2TT, UK; 3 School of Health and Related Research, University of Sheffield, 30 Regent Street, Sheffield, S1 4DA, UK; 4 General Practice and Primary Care Research Unit, University of Cambridge, Robinson Way, Cambridge, CB2 2SR, UK; 5 Oxford Centre for Diabetes, Endocrinology and Metebolism Churchill Hospital, Headington, Oxford, OX3 7LJ

## Abstract

**Background:**

We do not yet know how to use blood glucose self-monitoring (BGSM) most effectively in the self-management of type 2 diabetes treated with oral medication. Training in monitoring may be most effective in improving glycaemic control and well being when results are linked to behavioural change.

**Methods/design:**

*DiGEM *is a three arm randomised parallel group trial set in UK general practices. A total of 450 patients with type 2 diabetes managed with lifestyle or oral glucose lowering medication are included. The trial compares effectiveness of three strategies for monitoring glycaemic control over 12 months (1) a control group with three monthly HbA1c measurements; interpreted with nurse-practitioner; (2) A self-testing of blood glucose group; interpreted with nurse- practitioner to inform adjustment of medication in addition to 1; (3) A self-monitoring of blood glucose group with personal use of results to interpret results in relation to lifestyle changes in addition to 1 and 2.

The trial has an 80% power at a 5% level of significance to detect a difference in change in the primary outcome, HbA1c of 0.5% between groups, allowing for an attrition rate of 10%. Secondary outcome measures include health service costs, well-being, and the intervention effect in sub-groups defined by duration of diabetes, current management, health status at baseline and co-morbidity. A mediation analysis will explore the extent to which changes in beliefs about self-management of diabetes between experimental groups leads to changes in outcomes in accordance with the Common Sense Model of illness. The study is open and has recruited more than half the target sample. The trial is expected to report in 2007.

**Discussion:**

The *DiGEM *intervention and trial design address weaknesses of previous research by use of a sample size with power to detect a clinically significant change in HbA1c, recruitment from a well-characterised primary care population, definition of feasible monitoring and behaviour change strategies based on psychological theory and evidence, and measures along the hypothesised causal path from cognitions to behaviours and disease and well being related outcomes. The trial will provide evidence to support, focus or discourage use of specific BGSM strategies.

## Background

Diabetes is now a major public health problem. The number of people with diabetes is estimated to reach 330 million by 2030 [1]. There is a high burden from the disease: people with diabetes have an increased two to four fold risk of stroke and heart disease compared to the general population, and increased incidence of retinopathy, peripheral nerve damage and renal problems.

There is now strong evidence for the effectiveness of tight glycaemic control in reducing complications among people with diabetes [2]. However, the evidence with which to translate these research findings into guidance for delivery of health care is lacking [3]. In particular, efforts to promote self management of diabetes have shown limited and transient success in improving HbA1c levels [4]. Blood glucose self-monitoring (BGSM) is a technology that is frequently incorporated into self-management interventions, but has only been separately evaluated in a limited number of trials. Despite the lack of evidence, guidance is given both supporting and discouraging the use of BGSM.

BGSM was used to underpin insulin dose adjustment in the Diabetes Control and Complications Trial among people with type 1 diabetes, which clearly demonstrated the efficacy of glycaemic control in reducing diabetic complications. However, neither the rationale for BGSM nor its efficacy or effectiveness among people with type 2 diabetes is clear. Yet BGSM is now widely accepted as part of management of people with type 2 diabetes [5,6], and the costs associated with its use are rising rapidly [7]. Further trials are required to evaluate the benefit and cost-effectiveness of this technology and its place in the self management of people with type 2 diabetes.

### Target population

People with type 2 diabetes are at risk from a range of macrovascular and microvascular diabetic complications. Large trials have confirmed the effectiveness of intensive glycaemic control at reducing these complications [2]. Tight glycaemic control can be achieved through lifestyle change and medications. The target population will comprise the majority of patients on the average practice list with diabetes; we will focus on those patients within 5–10 yrs of diagnosis who are still comparatively healthy, of average age around 55–65 y, and managed on a range of medications and lifestyle advice. The exclusion of those with regular experience of BGSM will be required to avoid randomising them to a group not using meters.

### Limitations of previous research

#### Limited evidence for effectiveness from non randomised studies

A recent qualitative study has suggested that self-monitoring may be an important factor in helping people achieve a better understanding of their condition [8]. However, one study carried out in outpatient and general practitioner clinics in Italy found that increased frequency of monitoring was only associated with improved metabolic control in people able to adjust insulin doses [9]. A population study in the United States also found little relationship between testing frequency and HbA1c value [10]. By contrast, another large study using a cohort design carried out in a group model health maintenance organisation in California has suggested better glycaemic control among type 2 diabetes patients when using a BGSM compared to no use. Although attempts were made to control for differences between groups, the possibility of confounding between attitudes to self-care and use of BGSM cannot be excluded [11].

#### Limitations of and lack of evidence of effectiveness from randomised trials

Three systematic reviews have provided no evidence that self monitoring is effective in improving glycaemic control for people with type 2 diabetes when compared to urine testing and measurement of glycosylated haemoglobin (HbA1c) [12-14]. The majority of trials identified in these reviews have been carried out in small groups of people. Participants were not recruited from representative populations in the community and the strategies for use of the results from BGSM were not clearly defined. Two more recent studies, set both in hospitals and a family practice setting have adopted a more structured approach to relating blood glucose measurements to subsequent management decisions, but both trials have only published an analysis of people adhering to use of BGSM [15,16].

#### Research on mediators of effect not investigated in trials

There are a small number of studies that offer some insight into how BGSM might lead to improved blood glucose control among people with type 2 diabetes. BGSM may be helpful in the titration of therapy by either patients or by practitioners or both, but whether regular monitoring is more effective than periodic measurement of HbA1c is unknown. Evidence from qualitative studies with patients suggests that awareness of fluctuations in blood glucose levels may promote adherence to self-care behaviours, including medication taking, diet and physical activity in selected patients [8,17].

There is increasing work in the area of diabetes self-management that uses psychological theory to guide intervention and measurement of the processes of behaviour change. One approach, the Common Sense Model (CSM) [18], proposes that how people understanding threats to their health in central in determining efforts to minimise these health threats. For instance, if people with type 2 diabetes do not believe that physical activity affects their blood glucose levels, they have little reason to be more active to control their condition. Beliefs about illness can be categorised in terms of whether they relate to symptoms/ identity, cause, consequences, time lines, and control/ cure [18]. In support of the CSM, previous work has shown that beliefs about the consequences and controllability of diabetes, and the perceived effectiveness of treatment [19-21], predict patient adherence to recommended lifestyle management. Further, an intervention with myocardial infarction patients based on the CSM successfully managed to alter unhelpful beliefs, and led to faster return to work and fewer symptoms in the intervention group [23]. Further work using this approach to guide intervention and measures with people with type 2 diabetes may inform understanding of the potential mechanisms through which BGSM may improve health.

### Limitations of previous interventions

#### Technology

The majority of previous trials have used reflectance meters rather than biosensor technology. The older meters required larger quantities of blood and took longer to produce a reading than current systems. Although when used correctly the older meters provided reliable information, in practice their accuracy, usability and so potential impact was limited and may have formed a barrier to their effectiveness without high levels of motivation.

#### Strategy for use of meters

Only one identified randomised trial specified the approach used with patients to support their interpretation of test results [15]. Patients were told that using the meter and keeping records in a diary would provide information that would help them adjust their diet and lifestyle. A defined counselling algorithm was used to help ensure uniformity of delivery, but the extent to which the counselling helped patients relate the results to behaviour change is unclear. However, the impact of the intervention on self-perception, self-reflection and beliefs are not reported. The only published results of the study are from a per-protocol analysis rather than an intention to treat analysis [15]. Therefore, further work in which the intervention includes linking decision making to behaviour change is required.

#### Quality assurance

Previous trials have not specified the efforts made to monitor delivery of interventions. These include both the process of attempting to ensure that participants understand the techniques being used, and that efforts are made to ensure that the intervention is delivered as per protocol, and that there is adequate separation from the comparison groups in the intervention delivery.

### DiGEM Objectives

#### Primary objective

Our primary objective is to determine whether HbA1c is significantly lower in patients with type 2 diabetes allocated to each of two intervention groups (both receiving training in the techniques of blood glucose self-monitoring, but with one additionally receiving training in the interpretation and application of the results to diet, physical activity and medication adherence) compared to patients allocated to a control group (receiving standardised usual care involving intermittent measurement of HbA1c by health professionals).

#### Secondary objectives

Secondary objectives are:

(i) To compare well-being, satisfaction, health service use and economic cost between allocated groups.

(ii) To conduct an exploratory analysis of changes in mean HbA1c between regimens among sub-groups defined by duration of diabetes, current management, self-reported health status and co-morbidity.

(iii) To test how self-monitoring influences beliefs and behaviour using measures chosen on the basis of theoretical models of behaviour change.

## Methods/design

**Figure 1:**
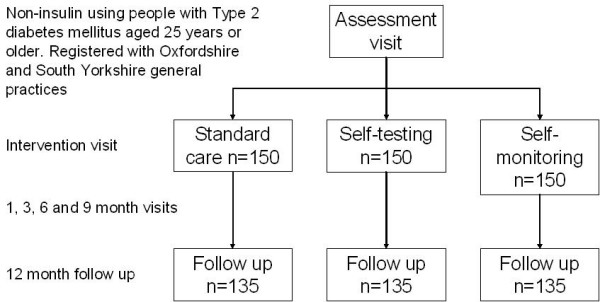
Consort diagram showing planned study numbers.

DiGEM is a four-year study with an open, randomised controlled pragmatic parallel group trial design (Figure 1) with sequential recruitment from two centres. The trial is managed from the Department of Primary Health Care, University of Oxford following NHS R&D Health Technology Assessment Programme guidelines. The study protocol was approved by the Oxfordshire B Research Ethics Committee.

The study design is shown in Figure 1. Participants are randomised to three groups consisting of

(i) a control group receiving standardised usual care and three monthly measurement of HbA1c,

(ii) a self-testing group who, in addition to the above, are carrying out blood glucose self testing with the results interpreted by the study nurse,

(iii) a self monitoring group who, in addition to both of the above, are given support in interpreting and applying the results of blood testing to enhance motivation and maintain adherence to diet physical activity and medication regimens.

Following randomisation, patients receive the allocated education and training appropriate for their group, with follow up to maintain the interventions at four, 13, 26, and 39 weeks with a final assessment at 52 weeks.

### Setting and recruitment

Non-insulin using patients with type 2 diabetes have been recruited from general practices in Oxfordshire and are being recruited from South Yorkshire to take part in the study. The 48 recruited practices represent a geographical spread of rural/suburban centres and cover a wide socio-economic range of patients. The mean number of patients recruited in the 24 practices in Oxfordshire was 10.2, standard deviation (SD) 5.6.

Patients suitable for trial inclusion are identified from practice generated computer lists. Eligible patients are sent an invitation to participate signed by their general practitioner accompanied by an information sheet and a reply paid envelope to facilitate response. One further letter is sent if no response is received in one month.

### Study population: inclusion and exclusion criteria

Inclusion criteria are type 2 diabetes, aged 25 years or more at diagnosis, managed with lifestyle or oral hypoglycaemic agents, independent for activities of daily living. Exclusion criteria are use of blood glucose monitor twice a week or more often over the previous three months, current use of insulin, co-morbidity or limited life expectancy that would make intensive glycaemic control inappropriate, last clinic HbA1c or HbA1c at the assessment visit less than 6.2%, or unable to follow trial procedures.

### Randomisation

Participants are randomly allocated to one of the three groups using a partial minimisation procedure to adjust the randomisation probabilities between groups to balance important covariates including duration of diabetes, HbA1c, and prior medication using a computer programme (Minim, ).

### Baseline measures and follow up

The primary outcome measure is change in HbA1c between the baseline measurement at the assessment visit and 12-month visit. Secondary outcome measures include change in systolic and diastolic blood pressure, weight, serum cholesterol and HDL, self-reported smoking status, dietary intake and physical activity (the Diabetes Self Care Activities Questionnaire) [22], medication adherence (The Medication Adherence Rating Scale) [23], and the scores in the Diabetes Treatment Satisfaction Questionnaire [24], and the Well-being Questionnaire (12 item) [25].

Beliefs about diabetes and its management are assessed using the Illness perceptions Questionnaire [26], Beliefs about Medicines Questionnaire [27], and a questionnaire developed for the study about the effectiveness of changes in eating and physical activity on the course of diabetes, and attitudes to blood glucose self-monitoring. Table 1 summarises the measures and their timing.

**Table 1:** Study measures

**Measures**	**Baseline**	**3 months**	**6 months**	**9 months**	**12 months**
**Questionnaire measures:**					
Illness perceptions questionnaire [26]					
Well-being questionnaire 12-item [25]					
Beliefs about medicines questionnaire					
Medication Adherence Report scale					
Beliefs about physical activity and eating +					
Beliefs about blood glucose monitoring +					
Beliefs about using a blood glucose monitor+					
Occupation and social class					
**Physiological measures**					
HbA1c					
Blood pressure					
Weight, height					
Total and HDL cholesterol					
**Costs**					
Use of medical services					
Costs of medication					
Costs of delivering intervention					

Blood glucose monitoring resources are measured by counting recorded entries in diaries. Medication use, episodes of hospitalisation and their duration will be noted and non-hospital health care resource use will be recorded by a questionnaire administered to all patients at each visit.

### Trial procedures

#### Assessment

Participant eligibility for the study and willingness to be randomised to a group in which they might be required to test their own blood glucose six times a week or more is confirmed at the assessment visit. Following consent, beliefs about diabetes, the role of eating, physical activity and medication are discussed with all participants. A goal setting approach to lifestyle change is introduced and continued in subsequent visits. Baseline blood tests and clinical measurements are taken and questionnaires completed at this visit.

Following the assessment visit and confirmation of eligibility on the basis of HbA1c measurement, patients are randomised to one of three groups: control group, self-testing and self-monitoring.

#### Post-randomisation

At a visit two weeks after the assessment visit participants receive training and education appropriate to their allocated study group. The control group receives 3-monthly HbA1c measurements and identifies behavioural goals to improve glycaemic control. The self-testing group, in addition, is asked to use a blood glucose meter to record three fasting, pre-meal or 2-hour post meal readings on two days during the week. Treatment targets of fasting and pre-meal levels of 4–6 mmol/l and post meal levels of 6 to 8 mmol/l and advice about using these readings to identify high (>15 mmol/l) and low (<4 mmol/l) blood glucose readings are given. The self-monitoring group, in addition, is provided with training and support to encourage interpretation of readings and application to goals for lifestyle change based on the CSM in order to reach treatment targets.

#### Follow up visits

Subsequent follow up includes a telephone call 2 weeks after randomisation (after the post-randomisation visit rather than randomisation), and further visits at 4, 13, 26 and 39 weeks. The follow up visits differ according to the allocated group. Those allocated to the control group have a HbA1c measure two weeks before their scheduled visit in order for their glycaemic control to be discussed. The two groups using a meter are managed on the results of their blood glucose self-monitoring. The GP is notified of all HbA1c results and asked to consider changes in medication in line with the National Institute for Clinical Excellence diabetes guidelines. The GP is also notified if blood glucose readings are consistently above 15 mmol/l.

#### Study measures (see Table 1 and Figure 1)

Baseline self-report measures and measures of belief are completed at the assessment visit. Baseline biochemical measures and clinical measurements are also made. Repeated measurements are made at the 52-week follow up visit. Data on adverse reactions or complications are collected at each study visit along with information about use of medication and health services.

#### Quality assurance and fidelity of interventions

Patients are supplied with a blood glucose meter calibrated to provide plasma equivalent results. Meters are checked at the beginning of the study and at 26 weeks by study nurses with a test aliquot.

A script outlining the topics to be covered, and explanation of the theoretical basis of the intervention are used to support the nurses in their adherence to study protocol. Study nurses attended a six day phased course in psychological theory, behaviour change techniques and skills training in the intervention. Additional measures to ensure fidelity include self-review of taped interventions by the study nurses and external review by a researcher using a checklist to ensure delivery of the intervention according to protocol. Prompts built into the patient diaries help participants adhere to their allocated group intervention.

#### Statistical aspects

In the absence of data relating to change in HbA1c we have calculated sample size conservatively based on the absolute level of HbA1c at follow up. We set out to detect a difference in HbA1c of 0.5% between any two groups. At the outset we estimated the SD of HbA1c as 1.5, based on data from the UKPDS. In practice, the SD of baseline HbA1c among the 245 patients recruited in Oxford was 0.9, but we assumed that the SD among additional patients recruited elsewhere could be as high as 1.5. With 205 further patients, the overall SD would be 1.2, and there would be 150 patients in each group (135 allowing for 10% attrition). These numbers would give 93% power to detect a difference of 0.5% in HbA1c between any two trial groups (2-tailed alpha = 0.05). Figure 1 gives estimates of likely numbers in each group and attrition.

We propose to conduct a single analysis of main trial endpoints at the end of the study. The proposed intention to treat analysis will compare mean levels of HbA1c at follow up between the three study groups, with baseline HbA1c as a covariate, using analysis of covariance. Post-hoc t-tests between groups will be conducted in the event of a statistically significant result. Subsequent analysis will include comparing the two intervention groups against the control group.

We will estimate the intervention effect in sub-groups defined by duration of diabetes (above or below median duration), current management (oral hypoglycaemic dugs or dietary management only), health status at baseline (above or below median EQ-5D score) and co-morbidity (presence or absence of diabetes related complications). We will also explore the extent to which the measures of beliefs included in the study can explain changes in behaviour; firstly by comparing mean levels of beliefs e.g. about controllability of diabetes between experimental groups and secondly by a more formal mediation analysis [28]. Within group analyses will be used to determine fidelity to protocol and conformity to the theoretical model, between group analyses will be used to assess impact of the intervention on key variables proposed by the theoretical model. Additional exploratory analysis will include changes in behaviour in relation to perceived threat and changes in perceived thereat from diabetes.

#### Economic evaluation

The economic evaluation will be on an intention-to-treat basis. A cost-effectiveness/cost-utility analysis will be performed in which the difference in effectiveness will be compared to the difference in total costs between each study intervention group, and the results will be expressed as incremental cost-effectiveness ratios. Effectiveness will be measured in terms of change in HbA1c, and modelled for life years gained and quality adjusted life years gained. Unit costs will be attached to the resource items collected from the healthcare resource utilisation assessment using published national average costs and tariff averages for procedures to calculate costs. Mean values and 95% confidence intervals will be reported for each component of resource use and cost and for total costs and effectiveness. Sensitivity analyses will be performed on all aspects of the economic evaluation that are subject to uncertainty.

## Discussion

This study will make an important contribution to the evidence-base for the use of blood glucose self-monitoring in non-insulin using patients with type 2 diabetes. It will provide a robust estimate of overall effect of use of the meters for self-testing alone and with a more intensive programme to support people in using their test results actively in self management of health related behaviours.

This trial will address two main problems associated with previous trials. Firstly, the study is adequately powered to identify a reduction in HbA1c of 0.5%, which is associated with clinically important reductions in diabetic complications. Secondly, study participants allocated to the most intensive group are explicitly supported in interpretation of their study results in relation to lifestyle changes. This is the first study in this field to use standardised delivery of these interventions together with the other measures supporting fidelity to protocol.

A further strength of the trial design is the inclusion of measures that explore the extent to which the interventions being used influences beliefs and behaviour. It will be possible to test whether the interventions, especially that based on the CSM, are successful in altering beliefs about diabetes, and whether the interventions were delivered with fidelity to their theoretical basis. It will also be possible to test whether differences in behaviour and HbA1c between experimental groups are due to the interventions bringing about changes in beliefs, as proposed by the CSM. Depending on the pattern of findings from the mediation analysis, it will be possible to conclude there is no mediation (none of the causal effect of intervention on behaviour is transmitted by beliefs about diabetes), total mediation (all of the effect is transmitted by beliefs) or partial mediation (part of the effect is transmitted by beliefs and part is direct). This will provide information about the extent to which the CSM can usefully inform intervention content to change beliefs and behaviours.

The trial is mainly generalisable to that group of patients willing to be randomised to no self-testing. It will be limited in its ability to inform management of people who are enthusiastic about regular meter use. Not only does this group include people who have already been recommended to use a meter by their doctor or nurse, but also includes people who have obtained a meter in the absence of medical advice. In health service terms, these are people who are likely to obtain a meter whether or not they are prescribed or offered one. A trial to address management in this group would be more difficult because of difficulties in identifying individuals who are both enthusiasts, yet willing not to be exposed to the use of self-monitoring. However, the detailed information about beliefs and the pre-specified sub-group analyses will provide information to inform design of future studies in this area by allowing refinement of interventions and accurate estimates to inform sample size calculations The detailed information from blood glucose diaries and relation to outcomes will also inform the refinement of training programmes and data interpretation.

This trial has features of both a pragmatic and an explanatory trial [29]. The use of three parallel groups, delivery of interventions according to protocol and extensive measures along a causal pathway are characteristic of phase III or explanatory trials. The use of a primary care setting, comparison of three feasible health service strategies, wide range of recruitment a long duration, and estimation of costs are, however, more characteristic of a pragmatic trial. This hybrid design, whilst answering a health service question, will provide additional information to guide future research. The extent to which the trial is able to deliver data that informs both of these agendas will inform design of trials of emerging health technologies where the opportunity to understand underlying mechanisms may be lost in an effort to simplify protocol design.

The results of this trial will be available in 2007.

## Competing interests

The authors hold no financial or non-financial competing interests. The views expressed in this paper do not necessarily reflect those of the NHS.

## Authors' contributions

AF, A-LK and AN had the original idea for the study and wrote the study protocol. AF, AW, DF and ALK developed study measures and intervention. AF, AW and LG have set up the study and made it run. AF is the guarantor of this paper.

## Investigators/TSC and DMEC

Members of the writing committee for this paper were A. Farmer, A. Wade, D French E Goyder HAW Neil, A-L Kinmonth.

The study investigators are Oxford: A Farmer, D Mant, R Holman, S Ziebland, R. Holman, A Gray, and P Yudkin (trial statistician); Cambridge A-L Kinmonth; Birmingham D French

Members of the trial steering committee are N Stott (Chair), A Farmer, HAW Neil, S Sutton, H Tewson, D Chapman, H Hearnshaw, E Goyder, and M Jiwa.

Members of the data monitoring committee are C. Baigent (Chair), J Levy and K Wheatley.

Coordinating Centres: (Oxford) A Wade (trial coordinator), A Craven (trial administrator), J Simon (health economist) and A Fuller (data manager); (Sheffield) Vivienne Walker

Study Nurses (Oxford) M Selwood, H Kirlow, M Chapman, and S Turner; (Sheffield) A Casbolt, K Dobson, and A Willert.

## Funding

Health Technology Assessment Programme and NHS R&D NHS Support Funding. AF is supported by an NHS R&D Career Development Award

## Pre-publication history

The pre-publication history for this paper can be accessed here:


